# *catena*-Poly[[di­aqua­bis­(4-formyl­benzoato-κ*O* ^1^)copper(II)]-μ-pyrazine-κ^2^ *N*:*N*′]

**DOI:** 10.1107/S1600536813032297

**Published:** 2013-12-07

**Authors:** Fatih Çelik, Nefise Dilek, Nagihan Çaylak Delibaş, Hacali Necefoğlu, Tuncer Hökelek

**Affiliations:** a Department of Chemistry, Kafkas University, 36100 Kars, Turkey; b Aksaray University, Department of Physics, 68100 Aksaray, Turkey; c Department of Physics, Sakarya University, 54187 Esentepe, Sakarya, Turkey; d Department of Physics, Hacettepe University, 06800 Beytepe, Ankara, Turkey

## Abstract

In the title polymeric compound, [Cu(C_8_H_5_O_3_)_2_(C_4_H_4_N_2_)(H_2_O)_2_]_*n*_, the Cu^II^ atom is located on a twofold rotation axis and has a slightly distorted octa­hedral coordination sphere. In the equatorial plane, it is coordinated by two carboxyl­ate O atoms of two symmetry-related monodentate formyl­benzoate anions and by two N atoms of the bridging pyrazine ligand, which is bis­ected by the twofold rotation axis. The axial positions are occupied by two O atoms of the coordinating water mol­ecules. In the formyl­benzoate anion, the carboxyl­ate group is twisted away from the attached benzene ring by 6.2 (2)°, while the benzene and pyrazine rings are oriented at a dihedral angle of 68.91 (8)°. The pyrazine ligands bridge the Cu^II^ cations, forming polymeric chains running along the *b*-axis direction. Strong intra­molecular O—H⋯O hydrogen bonds link the water mol­ecules to the carboxyl­ate O atoms. In the crystal, O—H_water_⋯O_water_ hydrogen bonds link adjacent chains into layers parallel to the *bc* plane. The layers are linked *via* C—H_pyrazine_⋯O_form­yl_ hydrogen bonds, forming a three-dimensional network. There are also weak C—H⋯π inter­actions present.

## Related literature   

For structural functions and coordination relationships of aryl­carboxyl­ate ions in transition metal complexes of benzoic acid derivatives, see: Nadzhafov *et al.* (1981 ▶); Shnulin *et al.* (1981 ▶). For applications of transition metal complexes with biochemical mol­ecules in biological systems, see: Antolini *et al.* (1982 ▶). Some benzoic acid derivatives, such as 4-amino­benzoic acid, have been extensively reported in coordination chemistry, as bifunctional organic ligands, due to the variety of their coordination modes, see: Chen & Chen (2002 ▶); Amiraslanov *et al.* (1979 ▶); Hauptmann *et al.* (2000 ▶). For a related structure involving 4-formyl­benzoate, see: Hökelek *et al.* (2009 ▶). For bond-length data, see: Allen *et al.* (1987 ▶).
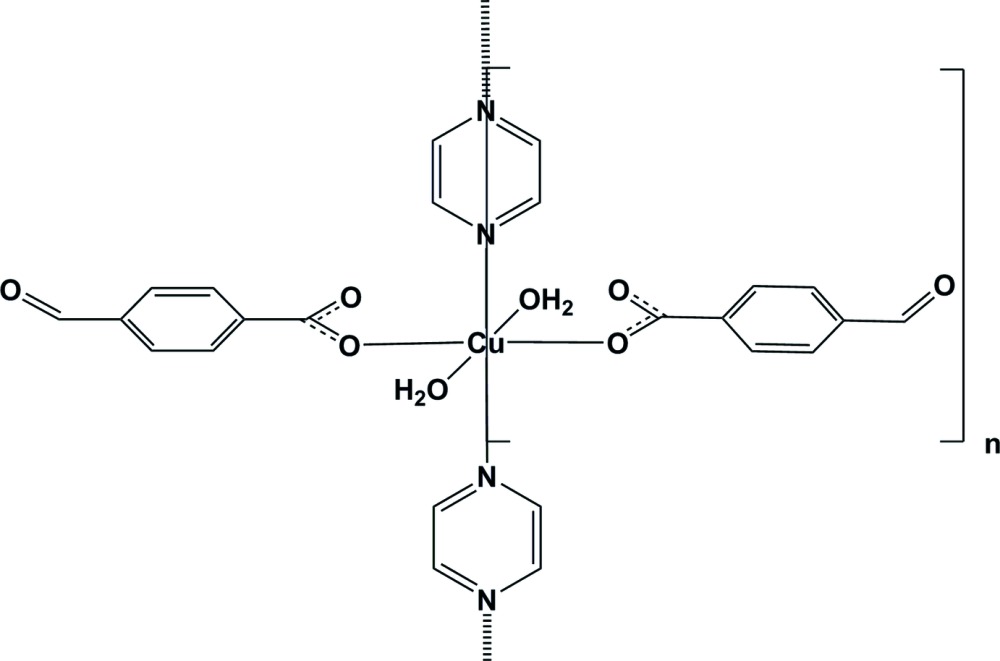


## Experimental   

### 

#### Crystal data  

[Cu(C_8_H_5_O_3_)_2_(C_4_H_4_N_2_)(H_2_O)_2_]
*M* *_r_* = 477.90
Monoclinic, 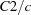

*a* = 21.7514 (4) Å
*b* = 6.8794 (2) Å
*c* = 12.9048 (3) Å
β = 93.621 (3)°
*V* = 1927.17 (8) Å^3^
*Z* = 4
Mo *K*α radiation
μ = 1.19 mm^−1^
*T* = 294 K
0.42 × 0.22 × 0.13 mm

#### Data collection  

Bruker SMART BREEZE CCD diffractometer
Absorption correction: multi-scan (*SADABS*; Bruker, 2012 ▶) *T*_min_ = 0.738, *T*_max_ = 0.857
14917 measured reflections
2398 independent reflections
2231 reflections with *I* > 2σ(*I*)
*R*_int_ = 0.029

#### Refinement  

*R*[*F* ^2^ > 2σ(*F* ^2^)] = 0.028
*wR*(*F* ^2^) = 0.089
*S* = 1.14
2398 reflections
150 parameters
2 restraints
H atoms treated by a mixture of independent and constrained refinement
Δρ_max_ = 0.47 e Å^−3^
Δρ_min_ = −0.30 e Å^−3^

### 

Data collection: *APEX2* (Bruker, 2012 ▶); cell refinement: *SAINT* (Bruker, 2012 ▶); data reduction: *SAINT*; program(s) used to solve structure: *SHELXS97* (Sheldrick, 2008 ▶); program(s) used to refine structure: *SHELXL97* (Sheldrick, 2008 ▶); molecular graphics: *ORTEP-3* for Windows (Farrugia, 2012 ▶); software used to prepare material for publication: *WinGX* (Farrugia, 2012 ▶) and *PLATON* (Spek, 2009 ▶).

## Supplementary Material

Crystal structure: contains datablock(s) I, global. DOI: 10.1107/S1600536813032297/su2669sup1.cif

Structure factors: contains datablock(s) I. DOI: 10.1107/S1600536813032297/su2669Isup2.hkl

Additional supporting information:  crystallographic information; 3D view; checkCIF report

## Figures and Tables

**Table 1:** Hydrogen-bond geometry (Å, °) *Cg* is the centroid of the C2–C7 ring.

*D*—H⋯*A*	*D*—H	H⋯*A*	*D*⋯*A*	*D*—H⋯*A*
O4—H42⋯O2	0.80 (2)	1.86 (2)	2.640 (2)	163 (3)
O4—H41⋯O4^i^	0.79 (2)	2.41 (3)	2.778 (2)	110 (3)
C9—H9⋯O3^ii^	0.93	2.49	3.335 (3)	152
C7—H7⋯*Cg* ^iii^	0.93	2.66	3.433 (2)	141

Symmetry codes: (i) 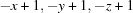; (ii) 
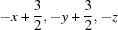
; (iii) 
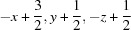
.

**Fig. 1.**
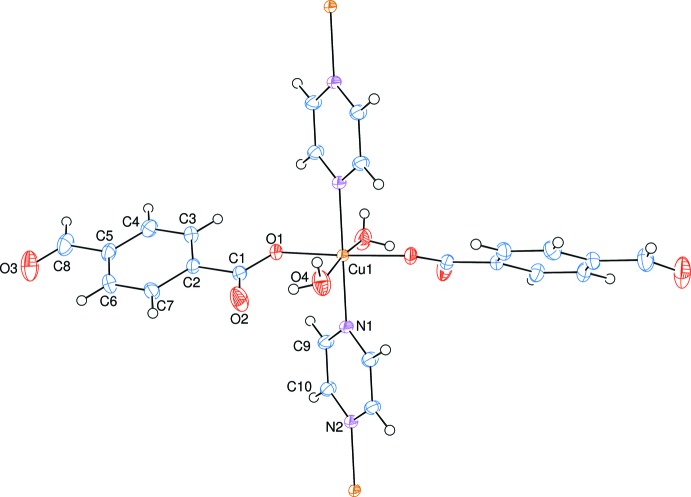
A view of the coordination geometry around the CuII atom of the title molecule, with atom labelling. Displacement ellipsoids are drawn at the 50% probability level. The two-fold rotation axis bisects atoms Cu1, N1 and N2.

**Fig. 2.**
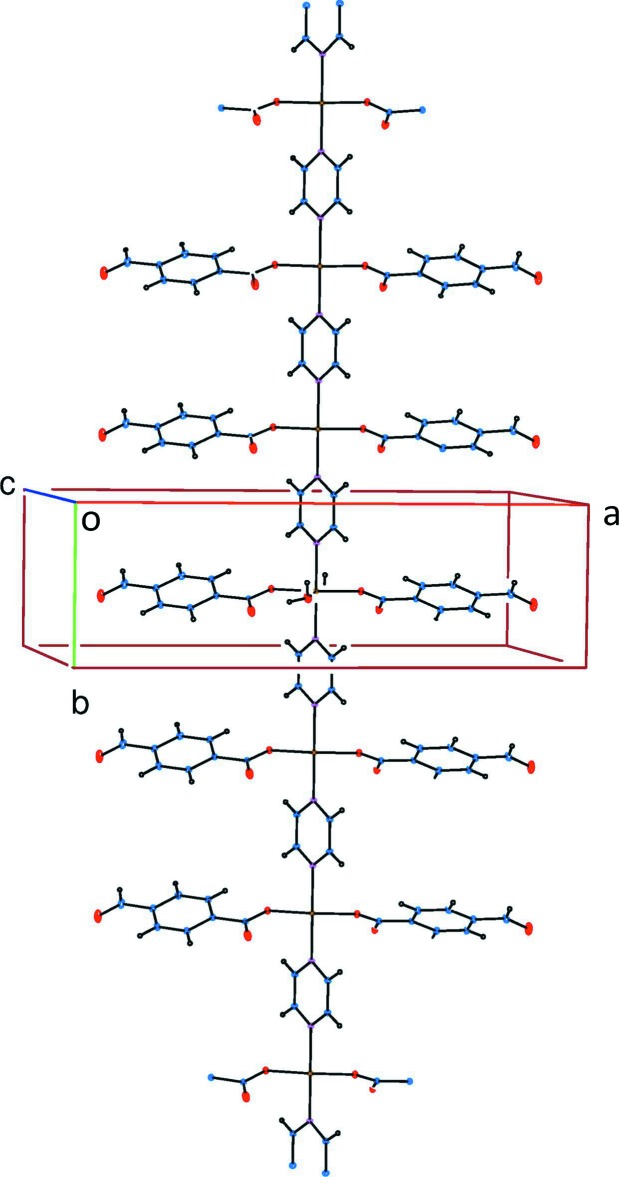
A partial view along the c axis of the crystal packing of the title compound.

## supplementary crystallographic information

### 1. Comment

The structural functions and coordination relationships of the arylcarboxylate ion in transition metal complexes of benzoic acid derivatives change depending on the nature and position of the substituent groups on the benzene ring, the nature of the additional ligand molecule or solvent, and the medium of the synthesis (Nadzhafov *et al.*, 1981; Shnulin *et al.*, 1981). Transition metal complexes with biochemically active ligands frequently show interesting physical and/or chemical properties, as a result they may find applications in biological systems (Antolini *et al.*, 1982). Some benzoic acid derivatives, such as 4-aminobenzoic acid, have been extensively reported in coordination chemistry, as bifunctional organic ligands, due to the varieties of their coordination modes (Chen & Chen, 2002; Amiraslanov *et al.*, 1979; Hauptmann *et al.*, 2000). The title compound was synthesized and its crystal structure is reported herein.

The asymmetric unit of the title compound contains half a Cu^II^ ion, one formylbenzoate (FB) anion, one water molecule and half of a pyrazine molecule. Atoms Cu1, and N1 and N2 of the pyrazine ligand, are located on a two-fold rotation axis (Fig. 1). The pyrazine ligands bridge adjacent Cu^II^ ions forming polymeric chains running along the *b*-axis direction (Fig. 2). The distances between the symmetry related Cu^II^ ions [Cu1···Cu1^i^; symmetry code (i) = x, y-1, z] is 6.879 (3) Å.

In the equatorial plane of the Cu^II^ coordination sphere is compossed of two carboxylate O atoms (O1 and O1^ii^; symmetry code: (ii) -x+1, y, -z+0.5) of two symmtery related monodentate formylbenzoate anions and two N atoms (N1 and N2^i^) of the bridging pyrazine ligand, which is bisected by the two-fold rotation axis. The axial positions are occupied by two O atoms (O4 and O4^ii^) of the coordinated water molecules.

The near equality of the C1—O1 [1.278 (2) Å] and C1—O2 [1.234 (2) Å] bonds in the carboxylate group indicate a delocalized bonding arrangement, rather than localized single and double bonds. The average Cu—N bond length is 2.0497 (19) Å, while the Cu—O bond lengths are 1.9546 (12) Å (for benzoate oxygen O1) and 2.4768 (12) Å (for water oxygen O4), close to standard values (Allen *et al.*, 1987). The dihedral angle between the planar carboxylate group (O1/C1/O2) and the adjacent benzene ring (C2—C7) is 6.2 (2)°, while the benzene and pyrazine rings are oriented at a dihedral angle of 68.91 (8)°. Strong intramolecular O—H···O hydrogen bonds (Table 1) link the water molecules to the carboxylate oxygens.

In the crystal, O-H_water_···O_water_ hydrogen bonds (Table 1) link adjacent chains into layers parallel to the bc plane. The layes are linked via C—H_pyrazine_···O_formyl_ hydrogen bonds forming a three-dimensional network. There also weak C—H···π interactions present.

### 2. Experimental

The title compound was prepared by the reaction of CuSO_4_.5H_2_O (1.25 g, 5 mmol) in H_2_O (250 ml) and pyrazine (0.40 g, 5 mmol) in H_2_O (30 ml) with sodium 4-formylbenzoate (1.72 g, 10 mmol) in H_2_O (100 ml) at room temperature. The mixture was filtered and set aside to crystallize at ambient temperature for several days, giving blue block-like crystals.

### 3. Refinement

The water H atoms (H41 and H42) were located in a difference Fourier map and freely refined. The C-bound H-atoms were positioned geometrically and constrained to ride on their parent atoms: C—H = 0.93 Å with *U*_iso_(H) = 1.2*U*_eq_(C).

### Crystal data

**Table:**

[Cu(C_8_H_5_O_3_)_2_(C_4_H_4_N_2_)(H_2_O)_2_]	*F*(000) = 980
*M**_r_* = 477.90	*D*_x_ = 1.647 Mg m^−^^3^
Monoclinic, *C*2/*c*	Mo *K*α radiation, λ = 0.71073 Å
Hall symbol: -C 2yc	Cell parameters from 9971 reflections
*a* = 21.7514 (4) Å	θ = 2.4–28.4°
*b* = 6.8794 (2) Å	µ = 1.19 mm^−^^1^
*c* = 12.9048 (3) Å	*T* = 294 K
β = 93.621 (3)°	Block, blue
*V* = 1927.17 (8) Å^3^	0.42 × 0.22 × 0.13 mm
*Z* = 4	

### Data collection

**Table:**

Bruker SMART BREEZE CCD diffractometer	2398 independent reflections
Radiation source: fine-focus sealed tube	2231 reflections with *I* > 2σ(*I*)
Graphite monochromator	*R*_int_ = 0.029
φ and ω scans	θ_max_ = 28.4°, θ_min_ = 1.9°
Absorption correction: multi-scan (*SADABS*; Bruker, 2012)	*h* = −28→27
*T*_min_ = 0.738, *T*_max_ = 0.857	*k* = −8→9
14917 measured reflections	*l* = −17→17

### Refinement

**Table:**

Refinement on *F*^2^	Primary atom site location: structure-invariant direct methods
Least-squares matrix: full	Secondary atom site location: difference Fourier map
*R*[*F*^2^ > 2σ(*F*^2^)] = 0.028	Hydrogen site location: inferred from neighbouring sites
*wR*(*F*^2^) = 0.089	H atoms treated by a mixture of independent and constrained refinement
*S* = 1.14	*w* = 1/[σ^2^(*F*_o_^2^) + (0.0478*P*)^2^ + 1.6638*P*] where *P* = (*F*_o_^2^ + 2*F*_c_^2^)/3
2398 reflections	(Δ/σ)_max_ = 0.001
150 parameters	Δρ_max_ = 0.47 e Å^−^^3^
2 restraints	Δρ_min_ = −0.30 e Å^−^^3^

### Special details

**Table:**

Geometry. Bond distances, angles etc. have been calculated using the rounded fractional coordinates. All su's are estimated from the variances of the (full) variance-covariance matrix. The cell esds are taken into account in the estimation of distances, angles and torsion angles
Refinement. Refinement of F^2^ against ALL reflections. The weighted R-factor wR and goodness of fit S are based on F^2^, conventional R-factors R are based on F, with F set to zero for negative F^2^. The threshold expression of F^2^ > 2sigma(F^2^) is used only for calculating R-factors(gt) etc. and is not relevant to the choice of reflections for refinement. R-factors based on F^2^ are statistically about twice as large as those based on F, and R- factors based on ALL data will be even larger.

### Fractional atomic coordinates and isotropic or equivalent isotropic displacement parameters (Å^2^)

**Table:**

	*x*	*y*	*z*	*U*_iso_*/*U*_eq_	
Cu1	0.50000	0.56167 (4)	0.25000	0.0226 (1)	
O1	0.58159 (6)	0.55545 (17)	0.19469 (11)	0.0283 (3)	
O2	0.63771 (7)	0.6755 (3)	0.33131 (11)	0.0478 (5)	
O3	0.90018 (8)	0.6186 (4)	0.03875 (15)	0.0643 (7)	
O4	0.54054 (7)	0.5818 (3)	0.43338 (12)	0.0429 (5)	
N1	0.50000	0.8590 (3)	0.25000	0.0222 (5)	
N2	0.50000	1.2632 (3)	0.25000	0.0228 (5)	
C1	0.63185 (7)	0.6123 (2)	0.24173 (13)	0.0255 (4)	
C2	0.68848 (7)	0.5984 (2)	0.18010 (13)	0.0233 (4)	
C3	0.68624 (8)	0.5098 (3)	0.08295 (13)	0.0278 (4)	
C4	0.73941 (8)	0.4934 (3)	0.02948 (13)	0.0311 (5)	
C5	0.79501 (8)	0.5656 (3)	0.07299 (15)	0.0298 (5)	
C6	0.79729 (8)	0.6554 (3)	0.16972 (14)	0.0322 (5)	
C7	0.74435 (8)	0.6718 (3)	0.22284 (13)	0.0286 (5)	
C8	0.85122 (10)	0.5472 (3)	0.01458 (19)	0.0433 (7)	
C9	0.52244 (8)	0.9602 (2)	0.17228 (13)	0.0263 (5)	
C10	0.52268 (8)	1.1618 (2)	0.17257 (13)	0.0265 (5)	
H3	0.64910	0.46150	0.05390	0.0330*	
H4	0.73790	0.43420	−0.03540	0.0370*	
H6	0.83440	0.70420	0.19860	0.0390*	
H7	0.74590	0.73200	0.28750	0.0340*	
H8	0.84820	0.47380	−0.04600	0.0520*	
H9	0.53820	0.89410	0.11700	0.0310*	
H10	0.53900	1.22800	0.11770	0.0320*	
H41	0.5464 (15)	0.485 (3)	0.465 (2)	0.065 (10)*	
H42	0.5738 (10)	0.609 (5)	0.414 (2)	0.064 (9)*	

### Atomic displacement parameters (Å^2^)

**Table:**

	*U*^11^	*U*^22^	*U*^33^	*U*^12^	*U*^13^	*U*^23^
Cu1	0.0165 (2)	0.0139 (2)	0.0382 (2)	0.0000	0.0076 (1)	0.0000
O1	0.0170 (5)	0.0258 (6)	0.0428 (7)	−0.0016 (4)	0.0076 (5)	−0.0036 (5)
O2	0.0327 (7)	0.0712 (11)	0.0413 (8)	−0.0153 (7)	0.0155 (6)	−0.0173 (7)
O3	0.0307 (8)	0.0990 (15)	0.0650 (11)	−0.0003 (9)	0.0176 (7)	0.0074 (11)
O4	0.0330 (8)	0.0558 (10)	0.0408 (8)	−0.0057 (7)	0.0086 (6)	0.0119 (7)
N1	0.0214 (8)	0.0149 (9)	0.0308 (9)	0.0000	0.0065 (7)	0.0000
N2	0.0206 (8)	0.0160 (8)	0.0322 (9)	0.0000	0.0055 (7)	0.0000
C1	0.0211 (7)	0.0212 (7)	0.0350 (8)	−0.0011 (6)	0.0079 (6)	0.0000 (6)
C2	0.0186 (7)	0.0219 (7)	0.0298 (8)	0.0006 (6)	0.0046 (6)	0.0021 (6)
C3	0.0215 (7)	0.0302 (8)	0.0316 (8)	−0.0002 (6)	0.0002 (6)	−0.0008 (7)
C4	0.0312 (9)	0.0340 (9)	0.0284 (8)	0.0045 (7)	0.0045 (7)	−0.0018 (7)
C5	0.0236 (8)	0.0307 (9)	0.0359 (9)	0.0038 (6)	0.0093 (7)	0.0044 (7)
C6	0.0204 (7)	0.0372 (10)	0.0391 (9)	−0.0049 (7)	0.0032 (6)	0.0000 (8)
C7	0.0245 (8)	0.0312 (9)	0.0305 (8)	−0.0054 (7)	0.0044 (6)	−0.0046 (7)
C8	0.0317 (10)	0.0525 (14)	0.0475 (11)	0.0065 (8)	0.0170 (9)	0.0027 (9)
C9	0.0301 (8)	0.0206 (8)	0.0293 (8)	0.0001 (6)	0.0114 (6)	−0.0016 (6)
C10	0.0298 (8)	0.0200 (8)	0.0308 (8)	0.0006 (6)	0.0111 (6)	0.0029 (6)

### Geometric parameters (Å, º)

**Table:**

Cu1—O1	1.9547 (13)	C2—C3	1.392 (2)
Cu1—O4	2.4766 (15)	C2—C7	1.397 (2)
Cu1—N1	2.046 (2)	C3—C4	1.388 (2)
Cu1—N2^i^	2.053 (2)	C4—C5	1.392 (3)
Cu1—O1^ii^	1.9547 (13)	C5—C6	1.391 (3)
Cu1—O4^ii^	2.4766 (15)	C5—C8	1.482 (3)
O1—C1	1.278 (2)	C6—C7	1.381 (2)
O2—C1	1.234 (2)	C9—C10	1.3869 (19)
O3—C8	1.196 (3)	C3—H3	0.9300
O4—H42	0.80 (2)	C4—H4	0.9300
O4—H41	0.79 (2)	C6—H6	0.9300
N1—C9^ii^	1.3382 (19)	C7—H7	0.9300
N1—C9	1.3382 (19)	C8—H8	0.9300
N2—C10	1.3382 (19)	C9—H9	0.9300
N2—C10^ii^	1.3382 (19)	C10—H10	0.9300
C1—C2	1.511 (2)		
			
O1—Cu1—O4	94.21 (5)	C3—C2—C7	119.60 (15)
O1—Cu1—N1	91.25 (4)	C1—C2—C7	119.15 (14)
O1—Cu1—N2^i^	88.75 (4)	C1—C2—C3	121.23 (14)
O1—Cu1—O1^ii^	177.49 (5)	C2—C3—C4	120.02 (16)
O1—Cu1—O4^ii^	85.93 (5)	C3—C4—C5	120.01 (16)
O4—Cu1—N1	86.80 (5)	C4—C5—C6	120.08 (16)
O4—Cu1—N2^i^	93.21 (5)	C4—C5—C8	119.21 (18)
O1^ii^—Cu1—O4	85.93 (5)	C6—C5—C8	120.71 (17)
O4—Cu1—O4^ii^	173.59 (7)	C5—C6—C7	119.87 (17)
N1—Cu1—N2^i^	180.00	C2—C7—C6	120.41 (16)
O1^ii^—Cu1—N1	91.25 (4)	O3—C8—C5	125.6 (2)
O4^ii^—Cu1—N1	86.80 (5)	N1—C9—C10	121.31 (16)
O1^ii^—Cu1—N2^i^	88.75 (4)	N2—C10—C9	121.45 (16)
O4^ii^—Cu1—N2^i^	93.21 (5)	C2—C3—H3	120.00
O1^ii^—Cu1—O4^ii^	94.21 (5)	C4—C3—H3	120.00
Cu1—O1—C1	126.09 (12)	C3—C4—H4	120.00
Cu1—O4—H41	119.0 (18)	C5—C4—H4	120.00
Cu1—O4—H42	89.4 (18)	C5—C6—H6	120.00
H41—O4—H42	104 (3)	C7—C6—H6	120.00
C9—N1—C9^ii^	117.30 (18)	C2—C7—H7	120.00
Cu1—N1—C9	121.35 (10)	C6—C7—H7	120.00
Cu1—N1—C9^ii^	121.35 (10)	O3—C8—H8	117.00
Cu1^iii^—N2—C10	121.42 (10)	C5—C8—H8	117.00
C10—N2—C10^ii^	117.16 (18)	N1—C9—H9	119.00
Cu1^iii^—N2—C10^ii^	121.42 (10)	C10—C9—H9	119.00
O1—C1—O2	125.98 (16)	N2—C10—H10	119.00
O2—C1—C2	118.42 (15)	C9—C10—H10	119.00
O1—C1—C2	115.60 (14)		
			
O4—Cu1—O1—C1	−20.13 (13)	O1—C1—C2—C7	174.99 (15)
N1—Cu1—O1—C1	66.75 (12)	O2—C1—C2—C3	173.20 (18)
N2^i^—Cu1—O1—C1	−113.26 (12)	O2—C1—C2—C7	−5.2 (2)
O4^ii^—Cu1—O1—C1	153.44 (13)	C1—C2—C3—C4	−177.90 (16)
O1—Cu1—N1—C9	39.77 (10)	C7—C2—C3—C4	0.5 (3)
O1—Cu1—N1—C9^ii^	−140.23 (10)	C1—C2—C7—C6	177.88 (16)
O4—Cu1—N1—C9	133.92 (9)	C3—C2—C7—C6	−0.6 (3)
O4—Cu1—N1—C9^ii^	−46.08 (9)	C2—C3—C4—C5	0.0 (3)
O1^ii^—Cu1—N1—C9	−140.23 (10)	C3—C4—C5—C6	−0.4 (3)
O4^ii^—Cu1—N1—C9	−46.08 (9)	C3—C4—C5—C8	−179.83 (19)
Cu1—O1—C1—O2	2.3 (2)	C4—C5—C6—C7	0.4 (3)
Cu1—O1—C1—C2	−177.93 (9)	C8—C5—C6—C7	179.77 (19)
Cu1—N1—C9—C10	−179.70 (12)	C4—C5—C8—O3	172.6 (2)
C9^ii^—N1—C9—C10	0.3 (2)	C6—C5—C8—O3	−6.8 (4)
Cu1^iii^—N2—C10—C9	−179.70 (12)	C5—C6—C7—C2	0.1 (3)
C10^ii^—N2—C10—C9	0.3 (2)	N1—C9—C10—N2	−0.6 (2)
O1—C1—C2—C3	−6.6 (2)		

Symmetry codes: (i) *x*, *y*−1, *z*; (ii) −*x*+1, *y*, −*z*+1/2; (iii) *x*, *y*+1, *z*.

### Hydrogen-bond geometry (Å, º)

Cg is the centroid of the C2–C7 ring.

**Table:**

*D*—H···*A*	*D*—H	H···*A*	*D*···*A*	*D*—H···*A*
O4—H42···O2	0.80 (2)	1.86 (2)	2.640 (2)	163 (3)
O4—H41···O4^iv^	0.79 (2)	2.41 (3)	2.778 (2)	110 (3)
C9—H9···O3^v^	0.93	2.49	3.335 (3)	152
C7—H7···*Cg*^vi^	0.93	2.66	3.433 (2)	141

Symmetry codes: (iv) −*x*+1, −*y*+1, −*z*+1; (v) −*x*+3/2, −*y*+3/2, −*z*; (vi) −*x*+3/2, *y*+1/2, −*z*+1/2.
